# Supplementary material for: Small RNA sequencing analysis of exosomes derived from umbilical plasma in IUGR lambs
Source: Commun Biol. 2023 Sep 15;6:943. doi: 10.1038/s42003-023-05276-1 (PMC10504244; doi:10.1038/s42003-023-05276-1)

Small RNA sequencing analysis of exosomes derived from umbilical plasma in IUGR lambs

Jiawei Lu[1], Huixia Li[1,3], Xiaomin Zheng[2,3], Yuan Liu[1] & Peng Zhao[1]

[1] College of Animal Science and Technology, Nanjing Agricultural University, Nanjing 210095, China. [2] Research Institute for Reproductive Medicine and Genetic Diseases, Wuxi Maternity and Child Health Hospital, Jiangsu Wuxi, 214002, China. [3] These authors jointly supervised this work: Huixia Li, Xiaomin Zheng. email: lihuixia@njau.edu.cn; 972514523@qq.com

Supplementary Table 1. Particle concentration, size, and protein concentration of umbilical plasma-derived exosomes (n = 3).

Groups	Particle concentration × 10^{11}	Size range D10 and D90 (nm)	Mode size (nm)	Mean size (nm)	Protein concentration (μg/μL)
CON	1.49 ± 0.71	86.0 – 188.9	130.00 ± 0.90 ***	139.95 ± 1.65 ***	1.20 ± 0.39
HS-IUGR	1.85 ± 0.45	74.7 – 180.2	122.60 ± 0.60	130.95 ± 0.05	1.18 ± 0.44

*** indicated $P < 0.001$.

Supplementary Table 2. Sequencing data quality of exosomes in CON and HS-IUGR groups (n = 3).

Sample	Reads_raw	Q20_raw	Q30_raw	Raw_GC	Reads_clean	Percentage	Q20_clean	Q30_clean	Clean_GC
C01	13,325,048	93.40%	87.20%	56%	8,393,464	62.99%	96.81%	92.54%	52%
C02	11,832,244	95.50%	90.35%	53%	9,759,889	82.49%	96.70%	92.14%	51%
C03	28,344,277	93.30%	87.31%	57%	19,568,298	69.04%	97.61%	94.06%	55%
H01	14,679,616	89.66%	83.10%	57%	8,618,473	58.71%	94.55%	89.94%	52%
H02	13,937,981	94.38%	88.75%	57%	8,660,504	62.14%	97.99%	94.72%	55%
H03	11,491,407	86.74%	80.79%	54%	9,232,640	80.34%	93.14%	88.67%	51%

Supplementary Table 3. Rfam library comparison statistics.

Sample	Cis-reg	lncRNA	others	rRNA	snoRNA	sRNA	qualified_reads	mapped_reads	map_rate
C01	925	57	199,293	65,624	71	1625	7,644,338	466,632	6.10%
C02	355	19	51,041	20,376	32	489	9,510,309	182,693	1.92%
C03	2922	220	689,269	368,353	93	3563	17,305,830	679,425	3.93%
H01	952	66	176,425	62,576	65	2207	7,829,078	609,742	7.79%

H02	1029	59	255,306	88,254	43	1489	7,617,520	300,411	3.94%
H03	715	44	375,601	147,763	35	839	8,343,022	310,383	3.72%

Supplementary Table 4. Nutritional composition of the silage.

Metabolic energy, MJ/kg	10.10
Crude protein, %	11.85
Calcium, %	0.62
Phosphorus, %	0.50
Neutral detergent fibre %	27.06
Acid detergent fibre, %	11.68

Supplementary Table 5. Primer sequences of miRNAs used for RT-qPCR.

miRNAs	Primer sequences (5'-3')
U6	F: AGCCTTCCACCTCAGCCTGTC
	R: TGGTCGGCAGTAAAGCAGAATCTC
oar-miR-411a-5p	RT: GTCGTATCCAGTGCAGGGTCCGAGGTATTCGCACTGGATACGACCGTACG
	F: CGCGCGATAGTAGACCGTATAG
	R: ATCCAGTGCAGGGTCCGAGG
oar-miR-200c	RT: GTCGTATCCAGTGCAGGGTCCGAGGTATTCGCACTGGATACGACCCATCA
	F: AAGCGCCTTAATACTGCCGGG
	R: ATCCAGTGCAGGGTCCGAGG

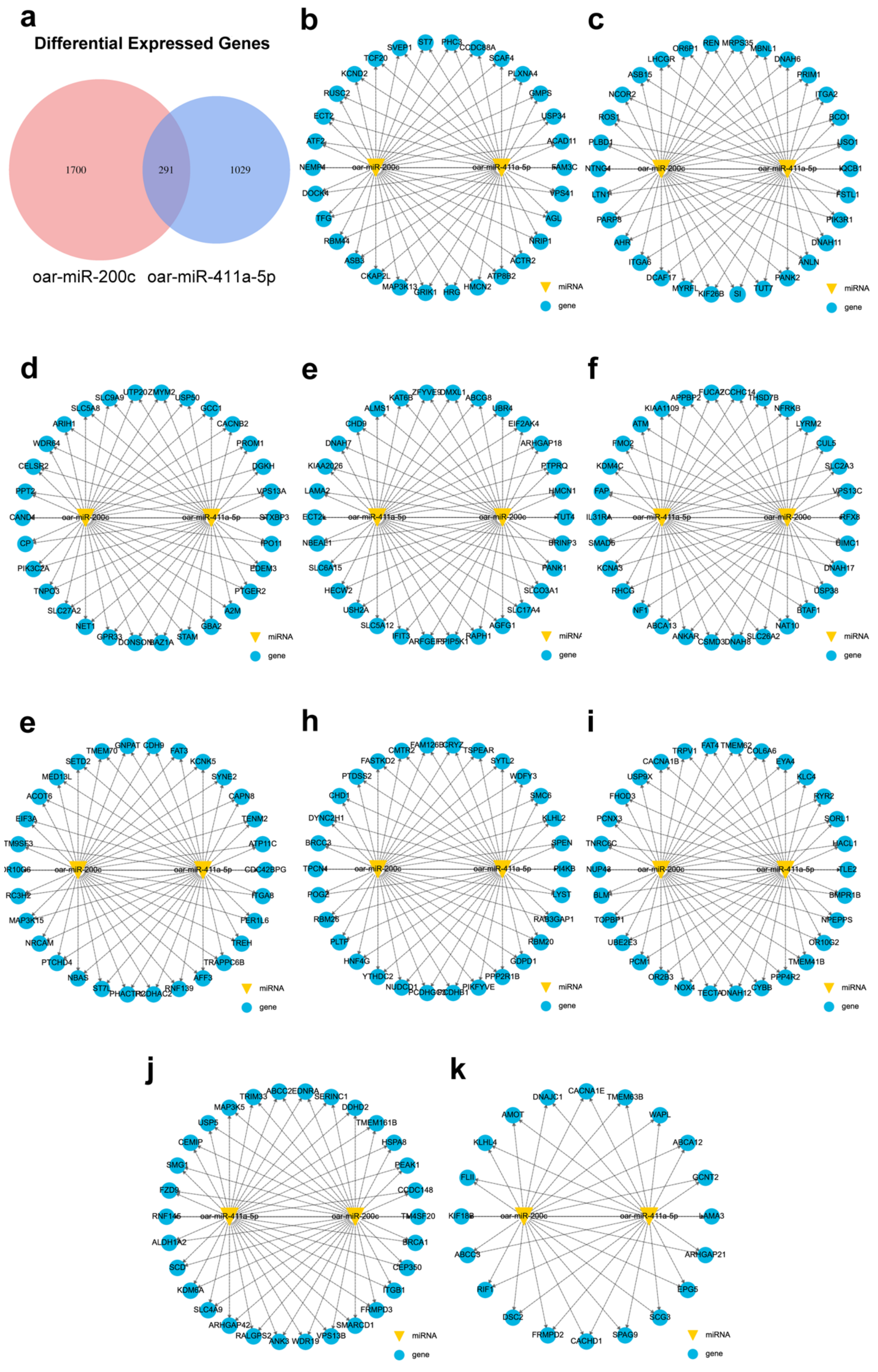

Supplementary Fig. 1 Predicted target genes of differentially expressed miRNAs

by miRanda. a Venn diagram shows the unique and common target genes of oar-miR-200c and oar-miR-411a-5p. **b-k** Common target genes of oar-miR-200c and oar-miR-411a-5p predicted by miRanda.

Western blot result of Calnexin.

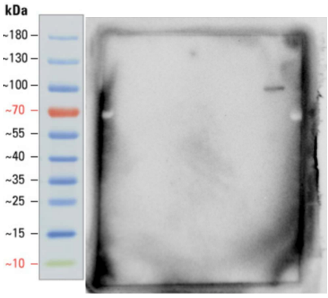

Western blot result of CD81.

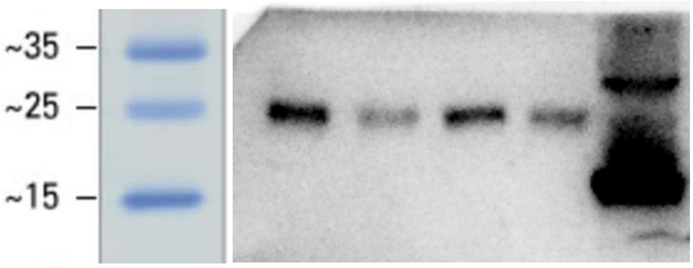

Supplement: Supplementary file 2 — Supplementary Information [file 42003_2023_5276_MOESM2_ESM.pdf]
